# Rapid, automated nerve histomorphometry through open-source artificial intelligence

**DOI:** 10.1038/s41598-022-10066-6

**Published:** 2022-04-08

**Authors:** Simeon Christian Daeschler, Marie-Hélène Bourget, Dorsa Derakhshan, Vasudev Sharma, Stoyan Ivaylov Asenov, Tessa Gordon, Julien Cohen-Adad, Gregory Howard Borschel

**Affiliations:** 1 SickKids Research Institute, Neuroscience and Mental Health Program, Hospital for Sick Children (SickKids), Toronto, ON Canada; 2 NeuroPoly Laboratory, Institute of Biomedical Engineering, Polytechnique Montreal, Montreal, QC Canada; 3 University of Toronto, Toronto, ON Canada; 4 Division of Plastic and Reconstructive Surgery, the Hospital for Sick Children, Toronto, ON Canada; 5 Functional Neuroimaging Unit, CRIUGM, University of Montreal, Montreal, QC Canada; 6 Mila - Quebec AI Institute, Montreal, QC Canada; 7 Indiana University School of Medicine, Indianapolis, IN USA

**Keywords:** Peripheral nervous system, Regeneration and repair in the nervous system

## Abstract

We aimed to develop and validate a deep learning model for automated segmentation and histomorphometry of myelinated peripheral nerve fibers from light microscopic images. A convolutional neural network integrated in the AxonDeepSeg framework was trained for automated axon/myelin segmentation using a dataset of light-microscopic cross-sectional images of osmium tetroxide-stained rat nerves including various axonal regeneration stages. In a second dataset, accuracy of automated segmentation was determined against manual axon/myelin labels. Automated morphometry results, including axon diameter, myelin sheath thickness and g-ratio were compared against manual straight-line measurements and morphometrics extracted from manual labels with AxonDeepSeg as a reference standard. The neural network achieved high pixel-wise accuracy for nerve fiber segmentations with a mean (± standard deviation) ground truth overlap of 0.93 (± 0.03) for axons and 0.99 (± 0.01) for myelin sheaths, respectively. Nerve fibers were identified with a sensitivity of 0.99 and a precision of 0.97. For each nerve fiber, the myelin thickness, axon diameter, g-ratio, solidity, eccentricity, orientation, and individual x -and y-coordinates were determined automatically. Compared to manual morphometry, automated histomorphometry showed superior agreement with the reference standard while reducing the analysis time to below 2.5% of the time needed for manual morphometry. This open-source convolutional neural network provides rapid and accurate morphometry of entire peripheral nerve cross-sections. Given its easy applicability, it could contribute to significant time savings in biomedical research while extracting unprecedented amounts of objective morphologic information from large image datasets.

## Introduction

Although peripheral nerve histomorphometry aims to quantify nerve fiber morphology precisely in research and clinical routine, commonly applied techniques require sampling and thereby fail to fully extract the rich, morphologic information included within the often thousands of nerve fibers per image.

**Figure 1:**
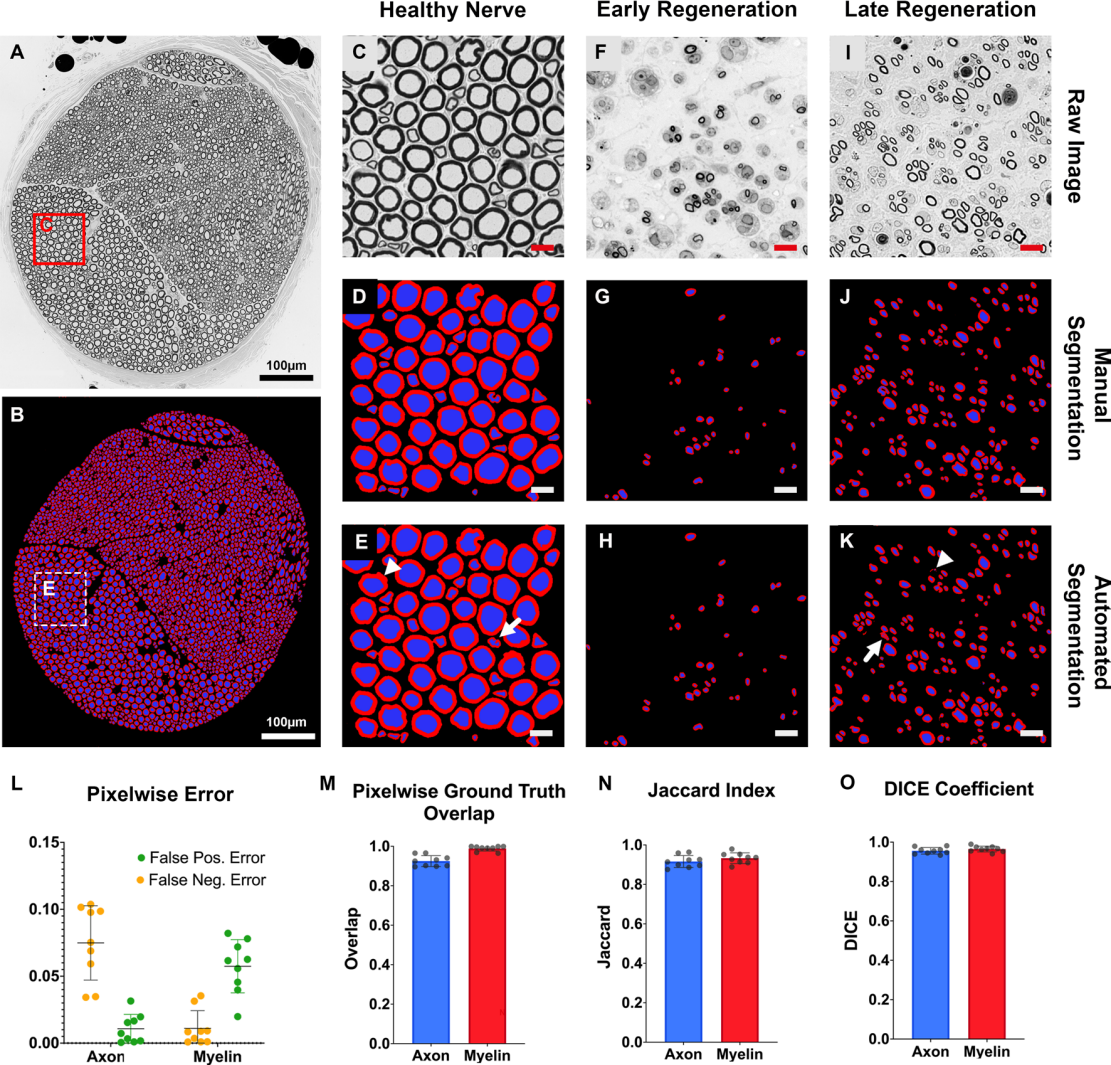
Pixelwise accuracy of automated axon/myelin segmentation. (**A**) Cross-section of a normal rat median nerve from the evaluation dataset. Myelinated axons are shown as black circles. Osmium tetroxide postfixed, epoxy embedded, 1 µm thickness. (**B**) Automated axon/myelin segmentation of the cross-section shown in A. (**C**) Magnified region of interest (ROI) from A. (**D**) Manual segmentation of myelin sheaths and axons of the ROI shown in C. (**E**) Automated segmentation of the ROI shown in C. Myelin sheaths are shown in red and axons are shown in blue in D and E. White arrows indicate segmentation inaccuracies due to processing artifacts of the myelin sheath (arrowhead) and irregularly shaped nerve fibers (white arrow). Scale bar represents 10 µm. (**F**) ROI from a rat common peroneal nerve in an early regeneration state, 3 weeks following nerve transection and epineural repair, 10 mm distal to the nerve repair site. (**G**) Manual and (**H**) automated segmentation of myelin sheaths and axons of the ROI shown in F. (**I**) ROI from a rat median nerve in a later regeneration state, 7 weeks following nerve transection and epineural repair, 10 mm distal to the nerve repair site. (**J**) Manual and (**K**) automated segmentation of myelin sheaths and axons of the ROI shown in I. White arrows indicate areas of over segmentation (arrowhead) and segmentation inaccuracies of singular nerve fibers with a complex shape (white arrow). (**L**) Pixelwise false positive and negative error for the automated axon and myelin segmentation compared to the ground truth. (**M**) Pixelwise overlap of the automated axon/myelin segmentations with the ground truth. (**N**) The Jaccard similarity index and (**O**) DICE coefficient indicate excellent similarity of the automated and manual axon and myelin segmentations. (own illustration created with AxonDeepSeg).

Axon diameter, myelin sheath thickness and g-ratio are key metrics for clinicians and researchers alike. They are frequently used for diagnosis and staging of neuropathic diseases^1–3^ and are commonly accepted outcome measures in nerve regeneration studies^4–6^. However, extremity nerves in common experimental rodent models include thousands of nerve fibers. Therefore, time consuming manual morphometry, although still frequently applied to this day, is usually limited to selected regions of interest (ROIs) and requires subsequent extrapolation. Due to the heterogenous distribution of morphologically and functionally distinct nerve fiber populations within peripheral nerves^7^ (Fig. 1A), the number of analyzed fibers and the size, number and location of selected ROIs may introduce considerable bias^8–10^. Even though methods for standardization such as systematic random sampling, have been proposed^11^, they too, are laborious, and their application has been reported inconsistently.

In recent years, a growing number of reports have aimed at developing computational methods for semiautomated and automated nerve fiber segmentation, in light- and electron-microscopy images of the central nervous system^12–15^ and peripheral nerve cross sections ^16–21^. However, the different size and shape of nerve fibers render their accurate automated detection and segmentation challenging. Following nerve injury regenerating axons are small, thinly myelinated, and thereby provide a low signal to noise ratio amongst intraneural debris. As a result, many laboratories have established custom-made methods for morphometry (personal communication), leading to poor methodological standardization among national and international research groups.

Neural networks offer the advantage of capable machine learning algorithms for pattern recognition in biomedical imaging data^22,23^. These models can be trained specifically for standardized and automated segmentation of large datasets and therefore, may be suitable for nerve fiber histomorphometry. AxonDeepSeg, is an open-source software library for axon/myelin segmentation across various microscopy modalities (https://axondeepseg.readthedocs.io/)^24^. Previous models were trained specifically for electron microscopic images^24^. Notably, their applicability was demonstrated in cross-sectional transmission electron microscopy images of the mouse striatum^25^ and tested in light microscopic images of peripheral nerves^26^. We aimed to extend the AxonDeepSeg framework by adding a model specifically trained for axon/myelin segmentation in light microscopic images of osmium tetroxide-stained rat peripheral nerve cross-sections. Here, we demonstrate its performance for nerve fiber segmentation and validate the automated histomorphometry in AxonDeepSeg against manual reference standards.

## Methods

This study is reported in accordance with the ARRIVE 2.0 guidelines (Animal Research: Reporting of In Vivo Experiments)^27^.

### Experimental animals

Tissue samples from 27 adult (200–300 g), female rats with a genetic Sprague Dawley background were included in this study. All animals were housed in a central animal care facility with fresh water and pellet food ad libitum. A constant room temperature (22 °C) and a circadian rhythm of 12 h per 24 h illumination were automatically maintained. All procedures were performed in strict accordance with the National Institute of Health guidelines, the Canadian Council on Animal Care (CCAC) and were approved by the Hospital for Sick Children’s Laboratory Animal Services Committee.

### Nerve samples

For this study, a total of 27 nerves from adult rats were used, all derived from previous and ongoing experimental nerve repair studies. Of these, 4 were tibial, 6 were median, and 17 were common peroneal nerves (Table 1). Surgical nerve harvesting procedures were performed under aseptic conditions and inhalation anesthesia with an Isoflurane (Baxter, Illinois, USA), oxygen mixture (3%, flow: 2 l/min). For analgesia, 4 mg/kg body weight extended-release Meloxicam (Metacam, Boehringer Ingelheim, Ingelheim, Germany) was injected subcutaneously. Nerves were harvested with a minimal touch technique from anesthetized animals. Regenerating nerves were harvested 7 to 10 mm distal to the nerve repair site (epineural repair with 10–0 non-absorbable monofilament suture). Samples were fixed in 4 °C precooled 2.5% glutaraldehyde (G6257, Sigma Aldrich) in pH 7.4 that was adjusted 0.1 M sodium cacodylate trihydrate buffer (C0250, Sigma Aldrich) overnight, followed by two 10 min washing steps in 0.1 M sodium cacodylate trihydrate buffer and, finally post-fixed in 2% osmium tetroxide (75,632, Sigma Aldrich) for 2 h at room temperature. The samples were dehydrated in ascending ethanol series, embedded in epoxy (45,345, Sigma Aldrich), sectioned into 1-μm cross-sections (ultramicrotome EM UC7, Leica Microsystems) and imaged (Axiovert 200 M, Carl Zeiss Microscopy GmbH, Jena, Germany) using a 63x/1.4 oil objective.

**Table 1:** Datasets for training and evaluation.

	Species	Nerve	Condition	Time post -repair (weeks)	Distance to nerve repair site (mm)	Tissue preparation	Embedding	Pixel size (µm)
*Training*	Rat	Tibial	Late Reg	12	7	2% OsO_4_ + TB	Epoxy	0.1
	Rat	Tibial	Late Reg	12	7	2% OsO_4_	Epoxy	0.1
	Rat	CP	Early Reg	3	10	2% OsO_4_	Epoxy	0.1
	Rat	CP	Early Reg	3	10	2% OsO_4_	Epoxy	0.1
	Rat	CP	Early Reg	3	10	2% OsO_4_	Epoxy	0.1
	Rat	CP	Early Reg	3	10	2% OsO_4_	Epoxy	0.1
*Testing*	Rat	CP	Early Reg	3	10	2% OsO_4_	Epoxy	0.1
	Rat	CP	Late Reg	7	10	2% OsO_4_	Epoxy	0.1
*Quality assessment*	Rat	CP	Early Reg	3	10	2% OsO_4_	Epoxy	0.1
	Rat	CP	Early Reg	3	10	2% OsO_4_	Epoxy	0.1
	Rat	CP	Late Reg	7	10	2% OsO_4_	Epoxy	0.1
	Rat	CP	Late Reg	7	10	2% OsO_4_	Epoxy	0.1
	Rat	CP	Late Reg	12	10	2% OsO_4_	Epoxy	0.1
	Rat	CP	Late Reg	12	10	2% OsO_4_	Epoxy	0.1
	Rat	CP	Late Reg	12	10	2% OsO_4_	Epoxy	0.1
	Rat	Tibial	Early Reg	3	7	2% OsO_4_ + TB	Epoxy	0.1
	Rat	Tibial	Early Reg	3	7	2% OsO_4_	Epoxy	0.1
	Rat	CP	Healthy	–	–	2% OsO_4_	Epoxy	0.1
Evaluation	Rat	Median	Healthy	–	–	2% OsO_4_	Epoxy	0.1
	Rat	Median	Healthy	–	–	2% OsO_4_	Epoxy	0.1
	Rat	Median	Healthy	–	–	2% OsO_4_	Epoxy	0.1
	Rat	Median	Late Reg	7	10	2% OsO_4_	Epoxy	0.1
	Rat	Median	Late Reg	7	10	2% OsO_4_	Epoxy	0.1
	Rat	Median	Late Reg	7	10	2% OsO_4_	Epoxy	0.1
	Rat	CP	Early Reg	3	10	2% OsO_4_	Epoxy	0.1
	Rat	CP	Early Reg	3	10	2% OsO_4_	Epoxy	0.1
	Rat	CP	Early Reg	3	10	2% OsO_4_	Epoxy	0.1

Training was performed on 6 light-microscopic cross-sectional images of osmium tetroxide stained, epoxy embedded rat nerves at various nerve regeneration stages. Another 12 images were used for testing and quality assessments. Performance of the trained model was evaluated using a separate image set including a total of 9 light-microscopic cross-sectional images of osmium tetroxide-stained epoxy embedded healthy and regenerating rat nerves.

*CP* common peroneal nerve, *Tibial* tibial nerve, *Median* median nerve, *Early Reg*. early nerve regeneration stage, *Late Reg*. late nerve regeneration stage, OsO_4_ osmium tetroxide, *TB* toluidine blue staining.

### Software and training process

AxonDeepSeg (version 3.2.0) was used for training the model. The AxonDeepSeg framework uses a four-step pipeline composed of data preparation, learning, evaluation, and prediction as previously described^24^. Briefly, for the data preparation step, light microscopy images and the corresponding manually segmented axon/myelin ground-truth masks were resampled to a common resolution of 0.1 μm/pixel and divided into patches of 512 × 512 pixels. For learning (training and testing), a dataset (Table 1) was used that included 8 images and ground-truth masks of entire rat nerve cross-sections (e.g. Fig. 5B) of various nerve regeneration stages. The manual ground-truth labelling process is described in the Manual Segmentations section. For the training, we used a U-net architecture^28^ with 3 convolutions per layer as previously described^24^. The model was trained for 400 epochs with a batch size of 7 and a learning rate of 0.005, using a Dice loss function^29^. To reduce overfitting, a dropout rate^30^ of 0.25 was used in convolutional layers, and a data augmentation strategy including random shifting, rotation, flipping and elastic deformation, was used on the input patches, and the masks as previously described^24^. The training was done on a GeForce GTX TITAN X GPU and took 9 h. The trained model was then used for inference on new microscopy images (prediction). Images were resampled, divided into patches, segmented, stitched to the native size, and resampled to the native resolution. Resampling was done using bilinear interpolation. For histomorphometry, AxonDeepSeg calculates the axon diameter from the cross-sectional area (A) of each axon assuming an ideal circular shape using the following equation:

$$Axon\;diameter = 2*\sqrt {\frac{A}{\pi }}$$

$$Myelin\;thickness = \frac{{\left( {2*\sqrt {\frac{{A_{axon} + A_{myelin} }}{\pi } } } \right) - Axon\;diameter}}{2}$$

### Prediction

#### Dataset

Performance of the trained model for prediction on new images was evaluated using a separate dataset (Table 1) including a total of 9 light microscopic cross-sectional images of healthy and regenerating rat nerves. Image pixel size was 0.1 µm. In each image, a region of interest (1008 × 1008 pixels = 1 016 064 pixels) was randomly selected and analyzed using automated segmentation in AxonDeepSeg, manual segmentation, and manual straight-line measurements to enable comparison between the three methods. Cropped nerve fibers at the edges of the ROIs, were excluded from the analysis.

#### Automated segmentations

The randomly selected ROIs were segmented automatically in axon/myelin mask using the “model_seg_pns_bf” in the AxonDeepSeg software. Depending on the brightness, raw images were preadjusted using the auto contrast function in Photoshop 2019 (version 20.0.0, Adobe, Mountain View, CA, USA). Regenerating nerves were segmented using a 1 × zoom with a 10-to-25-pixel overlap. Healthy control nerves were segmented with a 0.55 × zoom and a 10-pixel overlap to avoid over segmentation. Results of the subsequent automated histomorphometry were exported as Excel files. The analysis time was measured for each image from the start of the segmentation process until the segmentation overlay was completed.

#### Manual segmentations

GIMP (version 2.10) was used for manual segmentations. The inner and outer contours of the myelin sheath were carefully traced using the free select tool to obtain myelin labels. The region enclosed by the inner ring was subsequently filled to obtain axon labels. Histomorphometry of the manually created axon/myelin masks was conducted using the AxonDeepSeg algorithm. Analysis time was measured for each image from the start of the manual segmentation process until the segmentation overlay was completed.

#### Manual histomorphometry

The open-source platform ImageJ v 2.0.0^31^ was used for manual morphometry. Following scale setting, nerve fibers were counted and annotated using the multi-point tool. Axon diameters and myelin sheath thickness were measured for each individual nerve fiber using the straight-line tool. In non-circular shaped axons, the experimenter was instructed to measure a representative diameter that reflects the size of a circular nerve fiber. The results tab was exported to Excel. Analysis time was measured for each image from the start to completion of the annotation process.

#### Metrics

For determining the performance of automated segmentations created with AxonDeepSeg, the manually created axon/myelin labels were considered as ground truth. The pixelwise ground truth overlap, pixelwise false positive and negative error, and the DICE and Jaccard similarity indices were computed for axon and myelin masks respectively, using a MorphoLibJ plugin^32^ for ImageJ. For two binary images, a source image (automated segmentation) and a target image (ground truth mask), metrics were defined as follows:

$$Pixelwise\;ground\;truth\;overlap = \frac{{\left| {S \cap T} \right|}}{\left| T \right|}$$

$$Jaccard\;index = \frac{{\left| {S \cap T} \right|}}{{\left| {S \cup T} \right|}}$$

$$DICE\;coefficient = 2\frac{{\left| {S \cap T} \right|}}{\left| S \right| + \left| T \right|}$$

$$False\;negative\;error = \frac{{\left| {T/S} \right|}}{\left| T \right|}$$

$$False\;positive\;error = \frac{{\left| {S/T} \right|}}{\left| S \right|}$$

The performance of the automated nerve fiber detection in AxonDeepSeg was determined using precision, sensitivity, and F-Score. Precision, or the positive predictive value, determines the proportion of detected nerve fibers that was correct. Sensitivity (Recall) determines what proportion of nerve fibers present in the raw image was identified correctly. The F-score captures both precision and recall in a single metric. True positives were defined as axons present in both the automated segmentation and the ground truth mask. False positives were defined as axons present in the automated segmentation but absent in the ground truth mask. False negatives axons were defined as present in the ground truth mask but absent in the automated segmentation.

$$Precision = \frac{true\;positives}{{true\;positives + false\;positives}}$$

$$Sensitivity = \frac{true\;positives}{{true\;positives + false\;negatives}}$$

$$F\;Score = \frac{{\left( {2 * Precision * Sensitivity} \right)}}{{\left( {Precision + Sensitivity} \right)}}$$

### Statistical analysis

We used GraphPad Prism 9 (GraphPad Software, San Diego, California, USA) for statistical analysis. Descriptive statistics were calculated, and means are expressed with standard deviations (± SD). To test for normality of continuous variables, we used normal quantile plots and Shapiro–Wilk tests. For between group comparisons, one-way analysis of variance (ANOVA) was conducted with Tukey multiple comparison tests. Bland–Altman plots were used to plot agreement of automated histomorphometry (A) with morphometry obtained from ground truth labels (B) using a difference (B–A) vs average approach. A significance level of 5% was used (*p* < 0.05).

### Ethical approval

All procedures were performed in strict accordance with the National Institutes of Health guidelines, the Canadian Council on Animal Care (CCAC) and were approved by the Hospital for Sick Children’s Laboratory Animal Services Committee.

## Results

### Accuracy of automated axon/myelin segmentations with AxonDeepSeg

We determined the accuracy of automated segmentations in healthy (Fig. 1A–E) and regenerating nerves of early (Fig. 1F–H) and late regeneration stages (Fig. 1I–K) and compared them against manual axon/myelin labels (ground truths). Automated axon/myelin segmentations achieved pixelwise false negative and false positive errors below 0.1 respectively (Fig. 1L) and a mean pixelwise ground truth overlap of 0.93 ± 0.03 for axons and 0.99 ± 0.01 for myelin sheaths, respectively (Fig. 1M). Similarly, automated segmentations featured high similarity indices for axons and myelin sheaths of greater than 0.92 when compared to the ground truth (Fig. 1N and O). Subgroup analysis indicated comparable performance in early and late nerve regeneration stages and healthy nerves.

### Validation of automated nerve fiber histomorphometry with AxonDeepSeg

The AxonDeepSeg framework offers an integrated tool for automated nerve fiber histomorphometry based on axon/myelin segmentations. The morphometry results were created as an Excel file and include the total number of myelinated nerve fibers, their individual myelin sheath area and thickness, their axon area and diameter, the g-ratio, as well as metrics describing the nerve fiber’s shape. The latter includes solidity, eccentricity, orientation and their individual x and y coordinates. An image overlay is automatically created, enumerating all detected nerve fibers individually according to their number in the Excel sheet to facilitate manual correction of false negative and false positive nerve fibers. We validated the automated AxonDeepSeg histomorphometry by comparing against morphometry from manual ground truth labels (reference standard) and manual straight-line measurements with ImageJ.

### Nerve fiber count

**Figure 2:**
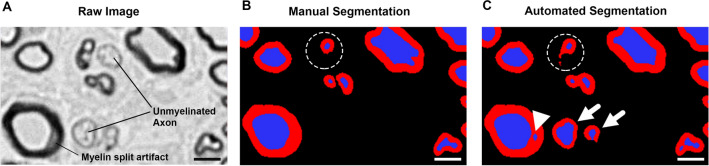
Automated axon identification. (**A**) Part of a cross-section of a rat tibial nerve in a late regeneration stage, 12 weeks post-surgery, 7 mm distal to the nerve coaptation. Myelinated axons are shown as black circles. Osmium tetroxide postfixed, epoxy embedded, 1 µm thickness. A myelin split artifact and prominent unmyelinated axons are present in this section. (**B**) Manual segmentation of the image in A by a blinded investigator. Myelin split artifacts and unmyelinated axons are correctly classified. Although an irregularly shaped, compressed nerve fiber is correctly identified as such, its myelin sheath is not fully segmented (dashed circle). (**C**) Automated segmentation of the image in A using AxonDeepSeg. The myelin split artifact (white arrowhead) as well as prominent unmyelinated axons (white arrows) have been misinterpreted as myelinated axons. Myelin sheaths are shown in red and axons are shown in blue. Scale bar represents 5 µm. (Own illustration created with AxonDeepSeg).

AxonDeepSeg detected a total of 1252 nerve fibers of which 1218 were true, achieving an overall precision of 0.97. The lowest precision of 0.96 was achieved in late nerve regeneration stages where 22 out of 665 detected nerve fibers were classified as false positives. These false positive nerve fibers were often attributable to either a misclassification of myelin split artifacts as separate axons (Fig. 2A,B, and C, white arrowhead), or to prominent unmyelinated nerve fibers that were misclassified as thinly myelinated nerve fibers (Fig. 2C, white arrows). Such misclassifications can be identified post hoc either by manual screening of the segmentation mask or by using the thresholding function in the Excel file containing the histomorphometry results. Misclassified myelin split artifacts usually feature very low “axon diameter” values combined with often very low “g-ratios”, whereas unmyelinated axons misclassified as myelinated axons are listed with atypically thin “myelin sheaths” and therefore unphysiologically high “g-ratios”.

Sensitivity analysis further revealed that AxonDeepSeg identified 99.4% of all myelinated nerve fibers present in the raw images. The highest sensitivity was achieved in early nerve regeneration stages with no false negative annotations. In late nerve regeneration stages, however, the automated segmentation missed 5 out of 648 nerve fibers (Table 2), leading to a slightly lower, though still excellent, sensitivity of 99.2% for this subgroup. To further quantify the performance of nerve fiber detection, we calculated the F-Score, representing the harmonic mean of precision and sensitivity. AxonDeepSeg achieved an overall F-score of 0.98 ranging from 0.97 in late regeneration stages to 0.99 in early regeneration stages, indicating excellent nerve fiber identification in healthy and regenerating nerves.

**Table 2:** The accuracy of automated axon segmentations using AxonDeepSeg in different nerve regeneration stages.

Group	Axons present	Axons detected	True positives	False positives	False negatives	Precision (POS. predictive value)	Sensitive (Recall)	F Score
Early Reg	154	157	154	3	0	0.98	1.0	0.99
Late Reg	648	665	643	22	5	0.96	0.99	0.97
Healthy	423	430	421	9	2	0.97	1.0	0.98
Overall	1225	1252	1218	34	7	0.97	0.99	0.98

### Axon diameter

**Figure 3:**
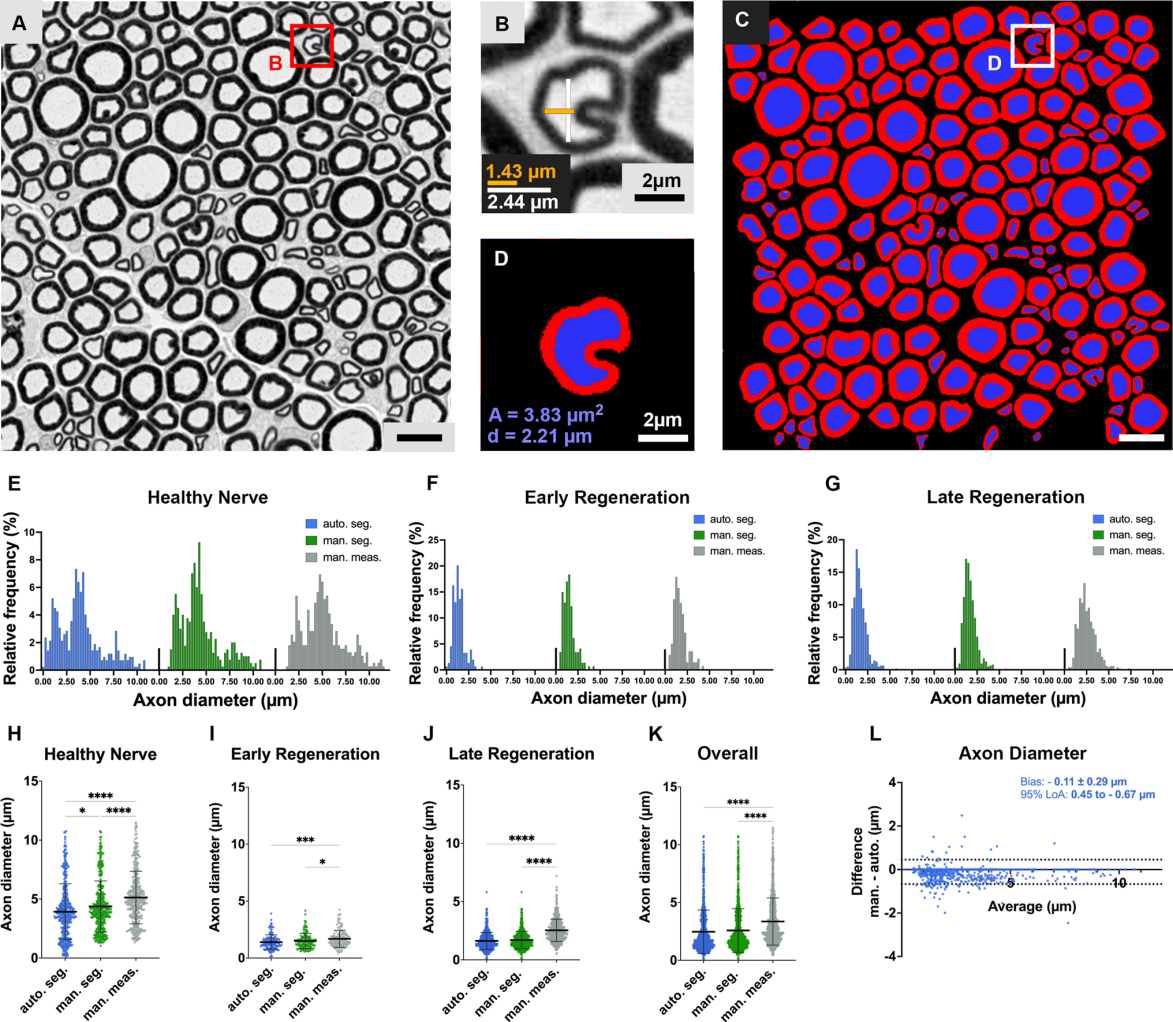
Automated axon diameter measurements. (**A**) Part of a cross-section of a normal rat median nerve. Myelinated axons are shown as black circles. Osmium tetroxide postfixed, epoxy embedded, 1 µm thickness, scale bar represents 10 µm. (**B**) Irregularly shaped nerve fiber. Axon diameter measurements using a straight-line technique can be unreliable as demonstrated for two different ways of measurements (orange = 1.43 µm; white = 2.44 µm) showing a 40% discrepancy. (**C**) Automated segmentation of A using AxonDeepSeg. (**D**) Automated segmentation of B. Myelin sheaths are shown in red and axons are shown in blue. To standardize axon diameter measurements, AxonDeepSeg calculates the axon diameter (**d**) from the cross-sectional area (**A**) of each axon assuming an ideal circular shape. (**E**) Histogram of the frequency distribution of nerve fibers according to their axon diameter in healthy nerves, (**F**) in early nerve regeneration stages 3 weeks post repair, and (**G**) in late nerve regeneration stages 7 weeks post repair. (**H**) Comparison of axon diameters obtained from automated segmentations (blue), ground truth labels (green) and manual morphometry (grey) for healthy nerves, (**I**) early nerve regeneration stages, (**J**) late regeneration stages and (**K**) all samples combined. When manually measured by an experimenter, the axon diameters were significantly larger compared to the ground truth histomorphometry, suggesting an observer bias towards selecting diameters that overestimate the axonal dimensions. (**L**) Nerve fiber specific comparison in a bland–Altman plot showing a good agreement of the AxonDeepSeg automated histomorphometry (auto.), with axon diameter ground truth measurements (man.). The bias represents the average of the differences and the 95% limits of agreement (LoA) comprise 95% of the differences between the two methods. (Own illustration created with AxonDeepSeg).

In experimental nerve repair studies, axon diameter measurements are critical to classify axons and to determine their maturity in regenerating nerves. To promote consistency among irregular nerve fiber shapes (Fig. 3A and B), AxonDeepSeg calculates the axon diameter from the cross-sectional area of each axon (Fig. 3C and D) assuming an ideal circular shape. When manually measured by an experimenter using a straight-line tool, the axon diameters were significantly larger compared to the ground truth histomorphometry, with mean difference of + 0.89 µm (Fig. 3E–K). This suggests an observer bias towards selecting diameters in often-non-circular axons that overestimate the axonal dimensions compared to the reference standard. Among all analyzed samples, the axon diameter calculated from automated segmentations were comparable to the diameter calculated from the ground truth with no significant differences between both methods (*p* > 0.05; Fig. 3K and L). Accordingly, Bland–Altman plots confirmed a high level of agreement for both methods (Fig. 3J). In healthy control nerves, however, subgroup analysis revealed a tendency of AxonDeepSeg to underestimate the axon diameter by 0.45 µm on average (*p* = 0.013, Fig. 3E and H). This was attributable mainly to misclassified myelin split artifacts as small axons as indicated by data points representing axons with a diameter close to zero in the blue scatter plot in Fig. 3H. In contrast, excellent agreement with the ground truth was achieved in early and late regenerating nerves (Fig. 3 F,G,I, and J).

### Myelin sheath thickness and g-ratio

**Figure 4:**
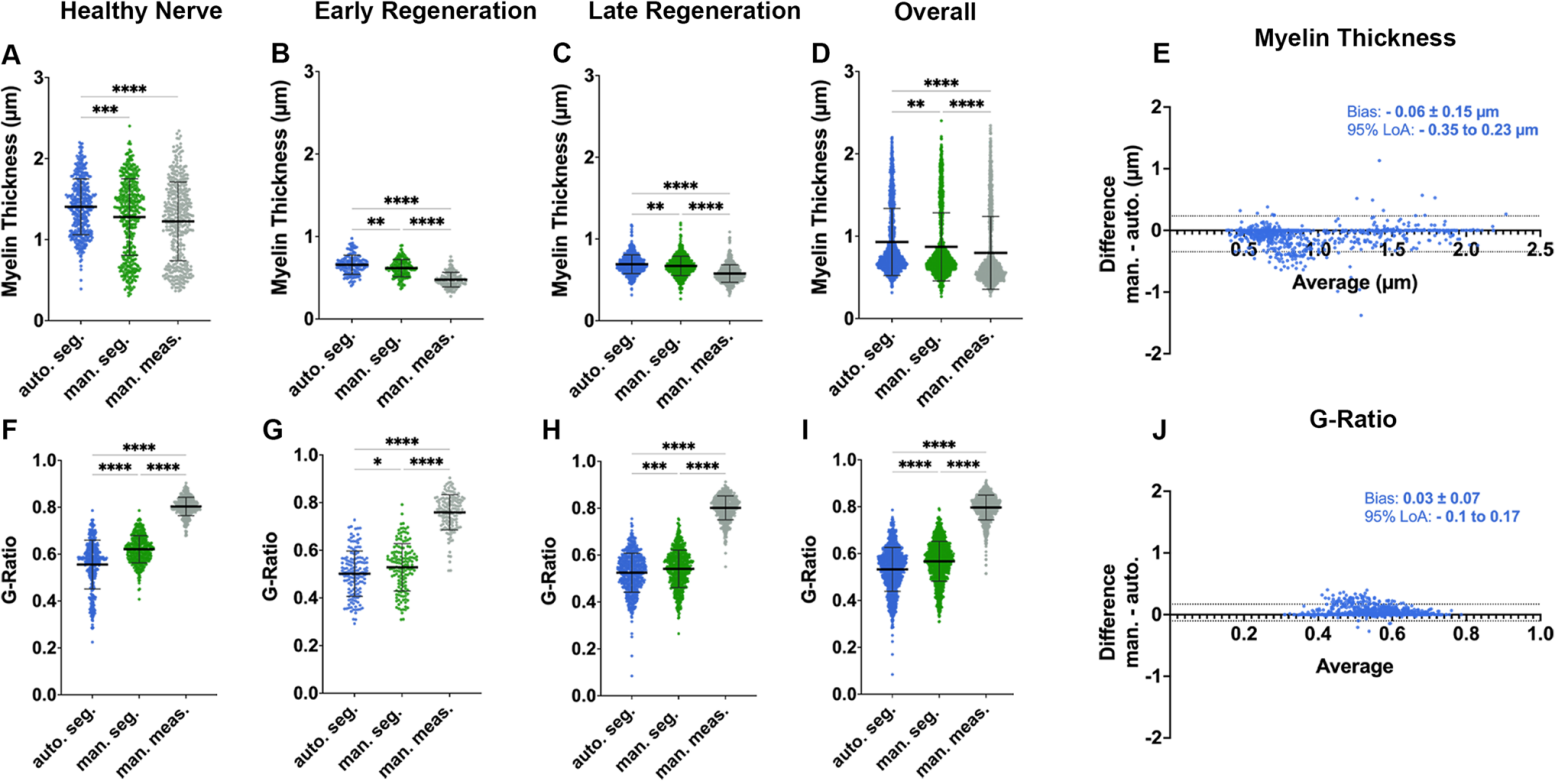
Automated myelin sheath thickness measurements and g-ratio calculations. (**A**) Comparison of myelin sheath thickness results obtained from automated segmentations (blue), ground truth labels (green) and manual morphometry (grey) for healthy nerves, (**B**) early nerve regeneration stages, (**C**) late regeneration stages and (**D**) all samples combined. (**E**) Nerve fiber specific comparison in a bland–Altman plot showing good agreement of the AxonDeepSeg automated histomorphometry (auto.), with myelin thickness measurements in the ground truth (man.). We observed a tendency of AxonDeepSeg to overestimate the myelin thickness by 0.06 ± 0.15 µm on average (bias) with the 95% limits of agreement (LoA) being -0.1 µm to 0.17 µm. (**F**) Comparison of the g-ratio calculations obtained from automated segmentations (blue), ground truth labels (green) and manual morphometry (grey) for healthy nerves, (**G**) early nerve regeneration stages, (**H**) late regeneration stages and (I) all samples combined. As a result of the axon diameter overestimation and underestimated myelin sheath thickness in manual measurements (grey), the g-ratios calculated from these metrics deviated drastically from the ground truth. (**J**) Nerve fiber specific comparison of the g-ratios in a bland–Altman plot showing an acceptable agreement of the automated histomorphometry via AxonDeepSeg (auto.) with the ground truth measurements (man).

Similar to the axon diameter, the myelin sheath thickness calculated in AxonDeepSeg is based on the cross-sectional area of each individual nerve fiber (myelin + axon) and the corresponding axon, both derived from automated segmentations (Fig. 3D). On average, the automated histomorphometry showed good agreement with the ground truth (Fig. 4A–E). However, we observed a tendency of AxonDeepSeg to overestimate the myelin thickness by 0.06 ± 0.15 µm on average compared to the reference standard (Fig. 4E). This phenomenon was particularly evident in healthy control nerves and is likely caused by the initial down-sampling of the raw image in this subgroup. Down-sampling aims at preventing over-segmentation by reducing image resolution. This may cause overestimation of the myelin thickness of small, thinly myelinated nerve fibers when the myelin sheath thickness comes close to, or even falls below the respective pixel size. This can be seen in the scatter plot in Fig. 4A when comparing the data points in green (ground truth) and blue (automated segmentation). Whereas the ground truth shows a distinct nerve fiber population featuring myelin sheaths below 0.8 µm, almost no nerve fibers with a corresponding myelin sheath thickness were detected by the automated algorithm. For regenerating nerves however, down-sampling was not required. Accordingly, for these subgroups we observed superior results of automated myelin sheath thickness measurements being closer to the ground truth when compared to manual morphometry (Fig. 4B and C).

As a result of the overestimated axon diameter and underestimated myelin sheath thickness obtained by manual straight-line measurements, the g-ratios calculated from these metrics deviated drastically from the ground truth (Fig. 4F–I, grey data). In contrast, g-ratios derived from automated histomorphometry showed a good agreement with the ground truth (Fig. 4J). However, misclassified false positive axons were identified again as a source of bias in automated segmentations as shown in Fig. 4F,H and I as a tail of data points corresponding to nerve fibers with untypically low g-ratios (< 0.35, blue data). Using manual post-hoc screening, experimenters can easily identify these outliers and thereby further improve the accuracy of automated histomorphometry.

#### Analysis time

**Figure 5:**
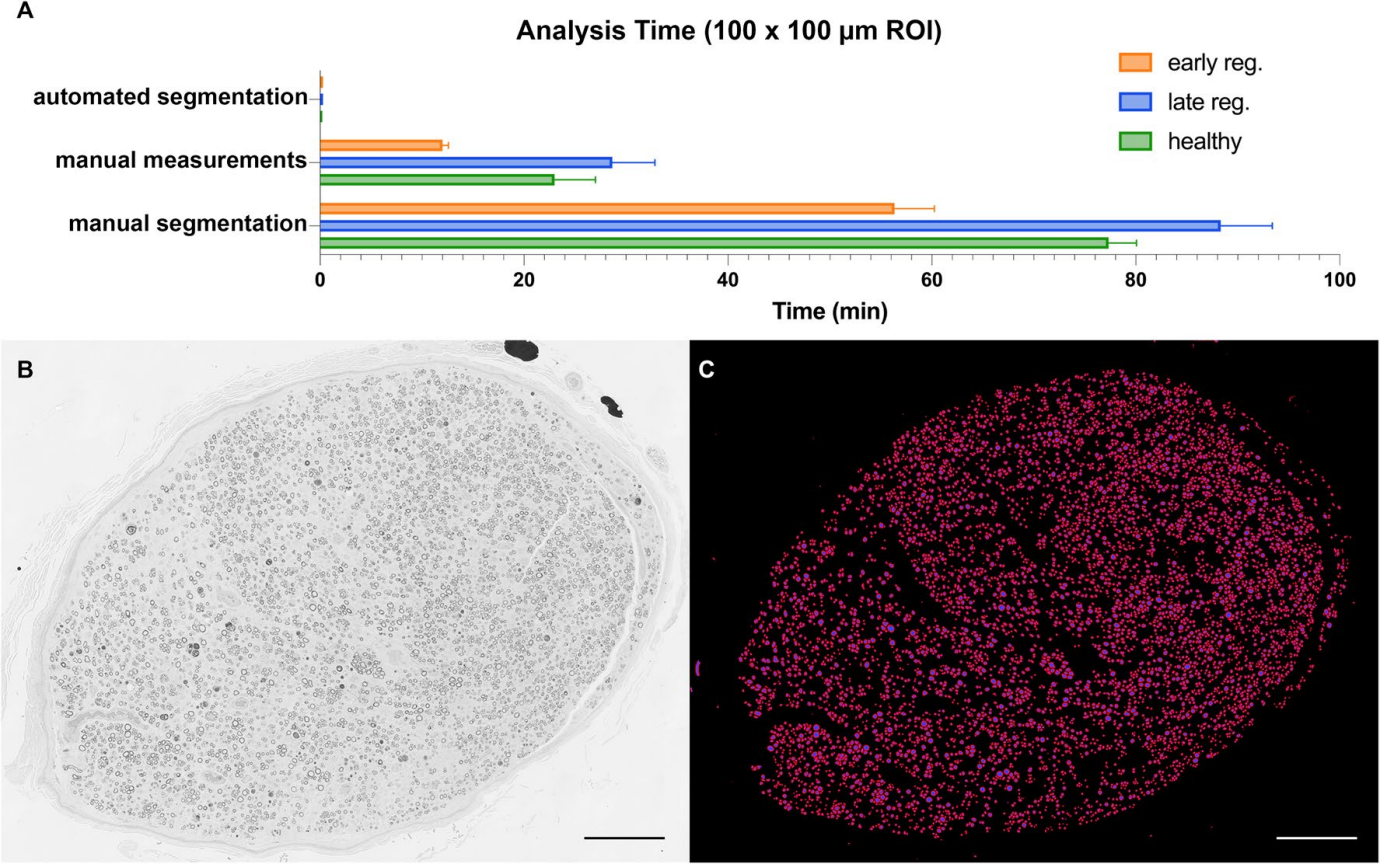
Analysis time. (**A**) Mean (± SEM) analysis times for a 100 × 100 µm region of interest in healthy nerves (green), early regeneration stages (orange) and late regeneration stages (blue) using automated segmentation with AxonDeepSeg, manual measurements with a straight-line tool in ImageJ or manual segmentations in GIMP. (**B**) To demonstrate the capabilities of AxonDeepSeg, we segmented an entire nerve cross-section of a rat median nerve 50 days after epineural nerve repair and 5 mm distal to the nerve repair site. Osmium tetroxide postfixed, epoxy embedded, 1 µm thickness. (**C**) The result of the segmentation included a total of 5894 myelinated nerve fibers after a computation time of 6 min and 11 s. The scale bars in B and C represents 100 µm. (Own illustration created with AxonDeepSeg).

Peripheral nerves often contain thousands of nerve fibers. Measuring all nerve fibers manually would require hours of work. Therefore, morphometry in large scale experiments is usually limited to selected regions of interest (ROI) and subsequent extrapolation. However, even for small ROIs manual morphometry is time- and resource-consuming. As shown in Fig. 5A, manual morphometry for a single 100 × 100 µm ROI required between 12 and 29 min of analysis time on average, depending on the regeneration stage and the number of axons present. Though highly accurate, manual segmentations, obtained by tracing the inner and outer contours of each myelin sheath, are even more time consuming (Fig. 5A) to a level that renders them obsolete for most large-scale research projects. In contrast, automated segmentation with AxonDeepSeg drastically reduced analysis time to between 13 and 18 s per ROI depending on the number of pixels (image resolution). This allows for morphometry of entire nerve cross-sections and thereby, overcomes the need for extrapolation and the associated risk of bias. In order to demonstrate the capabilities of the automated segmentation and morphometry we segmented an entire nerve cross-section of a rat median nerve 50 days after epineural nerve repair and 5 mm distal to the nerve repair site (Fig. 5B; CSA 0.4 mm^2^). Based on our results, we calculated that the manual morphometry of images in this regeneration stage would require approximately 8 s per nerve fiber. With a total of 5894 myelinated nerve fibers, complete manual morphometry of this nerve cross-section would have required 786 min of analysis time. In contrast, automated segmentation and morphometry took 6 min and 11 s (Fig. 5C; analysis was performed on a 2020 MacBook pro with a 10th gen. 2.0 GHz Quad‑Core Intel Core i5).

## Discussion

High resolution light microscopic images of nerve cross-sections often contain thousands of nerve fibers, each offering valuable morphologic information. However, effective approaches to fully extract this information for researchers and clinicians are presently unavailable because commonly applied methods often involve sampling^6,9–11^. Here we introduced and validated an open-source deep learning model that enables rapid automated nerve fiber segmentation and comprehensive morphometry in light-microscopic cross-sectional images of rat peripheral nerves.

The value and applicability of automated computational image analysis in research and clinical routine is defined by the ability to offer a close to human performance for a specific task. Neural networks are loosely inspired by the human brain and can learn to discriminate features from image data automatically in a process termed deep learning^33^. While early neural networks mostly offered subhuman performance, presently available algorithms are much more capable and may even surpass human performance in selected tasks^34,35^. As a result, imaging-intensive medical fields such as Radiology^23^, Ophthalmology^36^ and Dermatology^37^ have already started to implement artificial intelligence for clinical decision making with encouraging results. The histopathological analysis of peripheral nerves, although providing some of the most commonly reported metrics for experimental nerve studies, still relies on manual or semi-automated techniques that are time consuming, lack standardization and suffer from sampling bias^9–11,16,17,20^.

With this new model, we aimed at introducing a neural network to automatically segment axon/myelin and extract comprehensive morphologic information from light-microscopic cross-sectional images of peripheral nerves using AxonDeepSeg. Our results demonstrate that the model identified 99.4% of all myelinated nerve fibers with a positive predictive value of 0.97 in regenerating and normal nerves. Accurate extraction of morphologic information from each identified nerve fiber, such as myelin sheath thickness and axon diameter, then relies on precise delineation of the axon and its myelin sheath from the surrounding background. The model achieved pixelwise false negative and false positive errors below 0.1, with a pixelwise ground truth overlap of 0.93 for axons and 0.99 for myelin sheaths, respectively. Accurate axon/myelin segmentations were further corroborated by the results of the automated histomorphometry performed with AxonDeepSeg. In regenerating nerves, we compared the axon diameters of 822 axons from 9 different samples and found no significant difference between the automatic segmentations and the reference standard. We observed a minor tendency of the model to underestimate the axon diameter by 0.1 µm on average, mainly due to misclassified myelin split artifacts in healthy control nerves. Interestingly, this is in stark contrast to the results obtained with manual measurements of axon and myelin sheath thickness using a simple straight-line tool. Even though this method may be considered a historic gold standard for nerve fiber morphometry, we observed that this technique significantly overestimated the axon diameter by 0.89 µm on average. This highlights that often-non-circular nerve fibers cannot be accurately reflected by simple straight-line measurements (Fig. 3B) and suggests that observers may be inclined towards selecting diameters that overestimate the axonal dimensions compared to the reference standard. To account for irregular nerve fiber shapes and promote consistency, AxonDeepSeg standardizes these measurements by using the cross-sectional area of each nerve fiber to calculate axon diameter and myelin sheath thickness.

In addition to axon diameter, myelin thickness and g-ratio, AxonDeepSeg also extracts spatial information for each individual nerve fiber. This includes metrics describing the nerve fiber shape, such as solidity, eccentricity, and orientation as well as the individual x and y coordinates of each nerve fiber within a nerve cross section. By providing this comprehensive set of morphologic data on single-axon-resolution, AxonDeepSeg has the potential to amplify the amount of histological information extracted from each individual experimental unit. It further allows for precise spatial analysis of nerve fiber distributions within the nerve. For example, comparing nerve fiber populations from different regions of the nerve cross section may be valuable when assessing the penetration depth of locally delivered bioactive agents, identifying mechanical nerve entrapment, or characterizing neuropathic diseases.

Beyond performance, time and resource-efficiency is key for analysis methods applied in clinical routine and biomedical research. Although many laboratories still use manual analysis for critical samples, this simplified, almost technology-free approach to histomorphology is extremely time consuming, laborious, and inevitably fails in extracting most of the morphological information present in each slide. This is because a single nerve cross section requires hours of analysis time for an experienced analyst. As a result, analysis is often limited to a small sample of nerve fibers. Sampling comes with an inherent risk of bias^9^. For example, larger axons are more likely to be cropped at the edges of the sampling area which skews the results towards smaller fibers^8^. Further, differently sized axons are often distributed in clusters, with some areas of the nerve cross section having a higher percentage of large axons and other areas having a higher percentage of small axons^7,10,38^. Our results illustrate that the utilization of automated image analysis with AxonDeepSeg in biomedical research projects can drastically reduce the analysis time compared to manual measurements. This enables rapid analysis of larger datasets such as entire nerve cross-sections and thereby overcomes the need for sampling and the associated risk of sampling bias^3,9,10^. In conjunction with high-throughput whole-slide scanning technologies, tools such as AxonDeepSeg further allow for analysis of almost unlimited numbers of slides per nerve in experimental animals and human patients and therefore, may help to transform histomorphometric analysis from a laborious manual task to a component of big data precision medicine^39,40^.

Nowadays, capable commercial image analysis software often comes at high costs, thereby restricting access for many research laboratories. AxonDeepSeg can be downloaded free of charge and is compatible with most Mac, Linux, and Windows operating systems. Further, its open-source nature ensures full transparency and enables continuous performance enhancements and software extensions.

Although AxonDeepSeg is an improvement over existing methods for histomorphometry, it is not without limitations. Tissue processing artifacts such as myelin splitting, can cause misclassification as false positive axons. However, our results indicate that such misclassified axons usually feature a very small diameter close to zero and are therefore easily discernible from true positives with thresholding in the Excel sheet that contains the morphometry results. Further, we observed a tendency of AxonDeepSeg to slightly overestimate the myelin thickness in healthy control nerves. This phenomenon is likely caused by the required down-sampling of the raw image in this subgroup and may be a consequence of our training dataset that comprised regenerating nerves with thinly myelinated axons. Future work may therefore focus on further optimizing the performance of AxonDeepSeg’s models. This can be achieved by a continuous retraining of the neural network with an extended image dataset that includes samples from multiple laboratories to account for variability in sample processing, as well as samples from different species and neural pathologies. With increasing numbers of annotated training images, subtle features and differences between groups can be extracted by a neural network that may not even be apparent to the experimenter’s eye. We would therefore like to encourage colleagues from around the world to collaborate and participate in our efforts for the standardization of peripheral nerve histomorphometry in biomedical research by creating a capable freeware that truly achieves or even surpasses human performance. Another mitigation strategy against the reduced accuracy caused by down-sampling is to estimate the partial volume information in the output segmentation. This can be done using the SoftSeg algorithm which outputs a calibrated “soft” mask instead of a binary mask^41^.

In conclusion, the proposed AxonDeepSeg model enables rapid and automated axon/myelin segmentation and morphometry from light-microscopic images of peripheral nerve cross-sections with excellent precision and accuracy. Therefore, this open-source platform could contribute to significant time savings in experimental nerve research while extracting unprecedented amounts of quantitative, multiparametric morphologic information. Thus, although still in its infancy, neural network-based biomedical image analysis is likely to be a key technology for next-generation neuropathology.

## Data Availability

The data that support the findings of this study are available from the corresponding author upon reasonable request.

## Abbreviations

CP: Common peroneal nerve
TB: Toluidine blue staining
ROI: Region of interest
CACC: Canadian Council on Animal Care
S: Source image
T: Target image
ANOVA: One-way analysis of variance
